# Supplementary material for: Sialoglycans modulate Siglec-5-TLR4 interactions in osteoarthritis
Source: iScience. 2025 Nov 10;28(12):113979. doi: 10.1016/j.isci.2025.113979 (PMC12677102; doi:10.1016/j.isci.2025.113979)
Supplement: Document S1. Figures S1–S11 [file mmc1.pdf]

iScience, Volume *28*

Supplemental information

Sialoglycans modulate Siglec-5-TLR4 interactions in osteoarthritis

Loise Råberg, Fan Jia, Ula von Mentzer, Vignesh Venkatakrishnan, Niclas G. Karlsson, and Alexandra Stubelius

SUPPLEMENTAL INFORMATION

Figure S1: Gating strategy of monocyte populations (related to Figure 1)

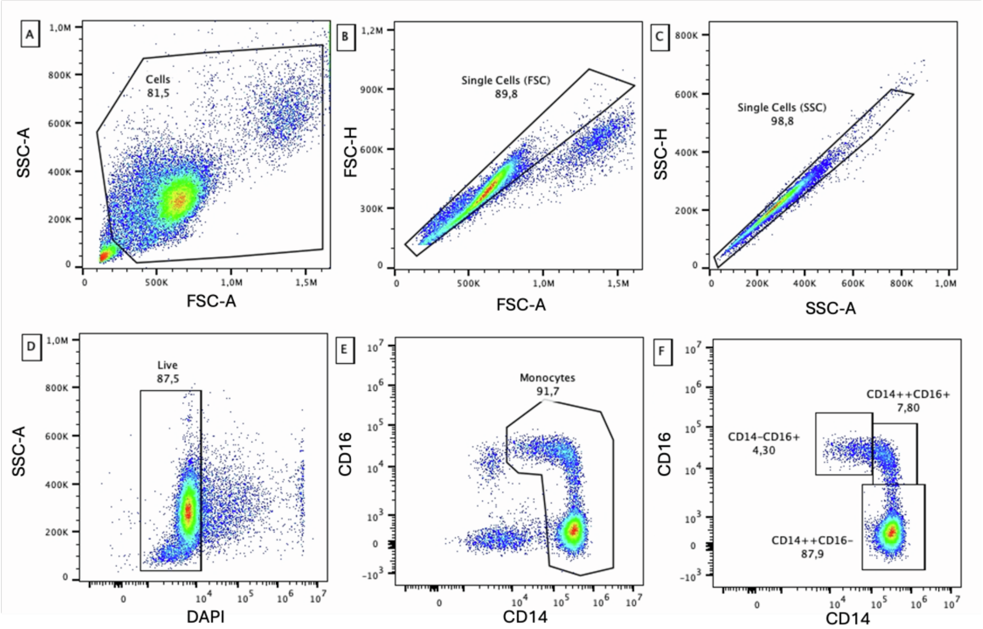

Figure S1: After isolating blood monocytes through negative selection, the cells were analyzed using flow cytometry. Debris was gated out using forward scatter area (FSC-A) versus side scatter area (SSC-A). Single cells were excluded first by putting FSC-A against FSC-H, followed by SSC-A against SSC-H. Live cells were detected by gating DAPI negative cells against SSC-A. Monocytic cells were gated out from the live cell population based on CD14 and CD16 expression, and non-monocytic cells were excluded. The ratio between classical ($CD14^{+}CD16^{-}$), intermediate ($CD14^{+}CD16^{+}$), and non-classical ($CD14^{dim}CD16^{+}$) was determined.

Figure S2: Monocyte isolation efficiency, percentage monocyte subsets, and expression of surface markers (related to Figure 1).

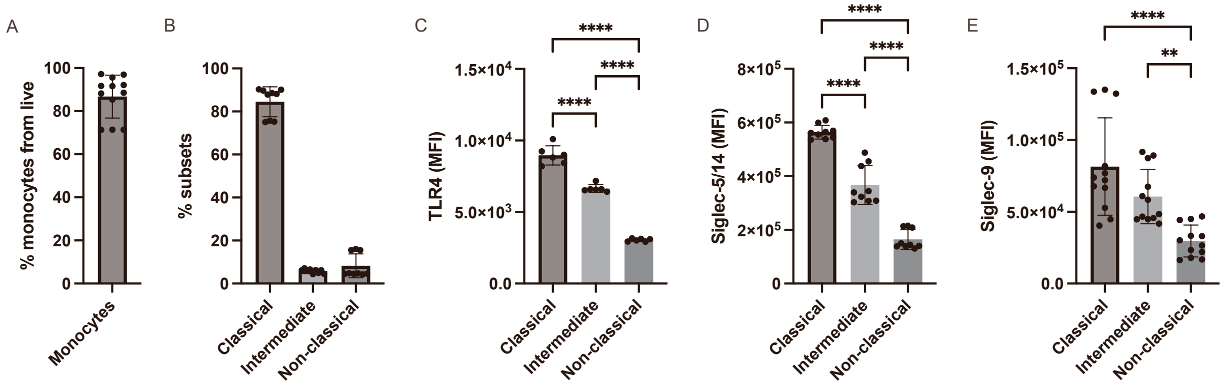

Figure S2: (A) The enriched monocyte fraction from the live cell population was confirmed to be between 72-85%, with a mean of 85.86%. (B) Percentage monocyte subsets at 0hrs. (C-E) Median fluorescence intensity (MFI) of TLR4, Siglec-5/14, and Siglec-9 in each monocyte subset at 0hrs. Data is represented as mean±SD with at least three technical replicates from three biological replicates. Statistical analysis was performed through one-way ANOVA using Dunnett's multiple comparisons test (*p<0.05; **p<0.01; ***p<0.001; ****p<0.0001).

Figure S3: Siglec-15 is not expressed by monocytes (related to Figure 1).

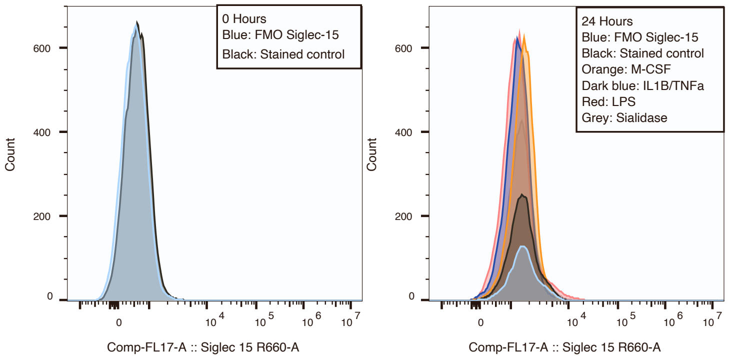

Figure S3: We investigated whether Siglec-15 was expressed at 0 and 24hrs but found no evidence of any membrane-associated Siglec-15 expression at these timepoints. The stained samples were compared to the Siglec-15 fluoresence minus one control (FMO).

Figure S4: Correlation analysis after OA SF stimulation (related to Figure 1).

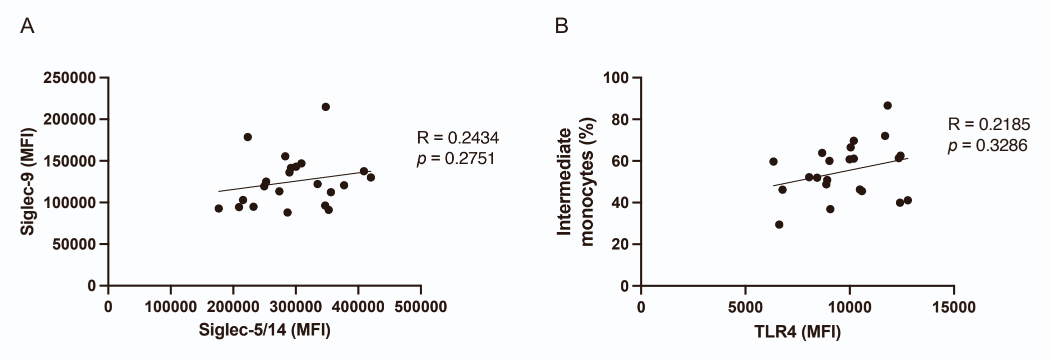

Figure S4: Correlation analysis was performed between Siglec-9 and Siglec-5/14 (A) and intermediate monocytes and TLR4 (B) after 24hrs of stimulation with OA SFs. Each data point represents the mean of three technical replicate samples from three biological replicates. Statistical analysis was performed through Spearman's correlation analysis (*p<0.05; **p<0.01; ***p<0.001; ****p<0.0001)

Figure S5: Expression of intermediate monocytes, TLR4, Siglec-5/14, and Siglec-9 at 4hrs of stimulation (related to Figure 2).

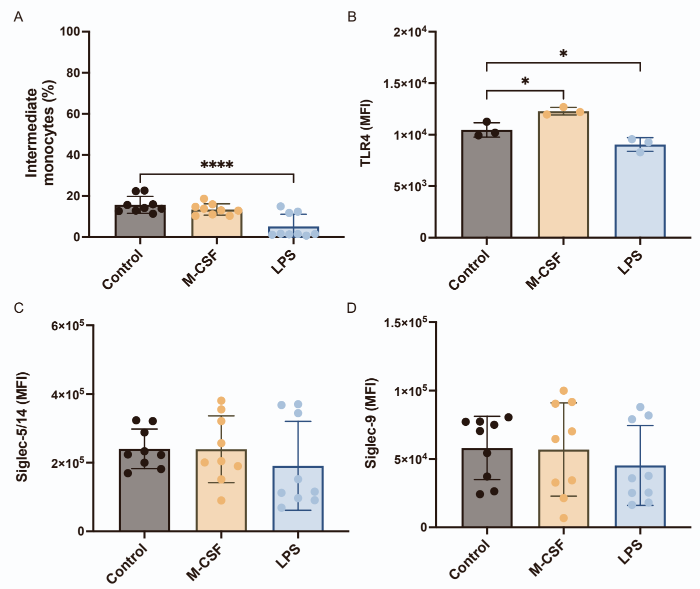

Figure S5: The blood monocytes were stimulated with M-CSF and LPS for 4hrs. The percentage intermediate monocytes, based on $CD14^{+}CD16^{+}$ (A), as well as MFI of TLR4 (B), Siglec-5/14 (C), and Siglec-9 (D). Data shown are representative of three biological replicates performed in triplicate, except TLR4, which was performed in one biological replicate. Statistical analysis was performed through one-way ANOVA using Dunnett's multiple comparisons test (*p<0.05; **p<0.01; ***p<0.001; ****p<0.0001).

Figure S6: Expression of TLR4 and Siglec-5 at 24hrs of stimulation with IL1β and TNFα (related to Figure 2).

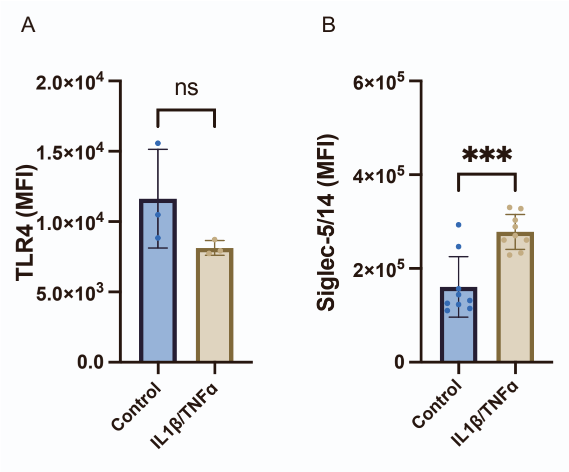

Figure S6: The MFI of TLR4 (A) and Siglec-5/14 (B) on monocytes was quantified after 24hrs of stimulation. Data is represented as mean±SD from three technical replicates from one biological replicate for TLR4 and three for Siglec-5. Statistical analysis was performed through unpaired t-test using (*$p<0.05$; **$p<0.01$; ***$p<0.001$).

Figure S7: In-silico protein-protein prediction in AlphaFold3 for direct contact between TLR4 and Siglec-5 (related to Figure 3).

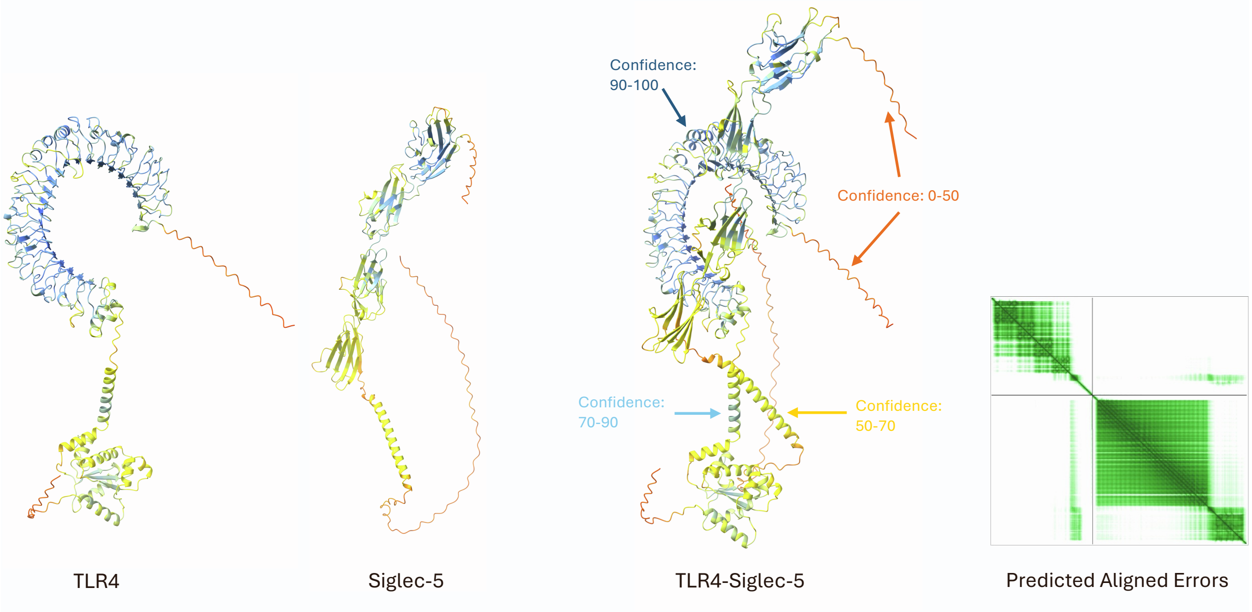

Figure S7: The predicted interaction between of TLR4 (O00206) and Siglec-5 (O15389) colored by pLDDT (orange, 0-50; yellow, 50-70; cyan, 70-90; blue, 90-100), were blue indicates high confidence in the prediction and orange color indicates low confidence. The Predicted Alignment Errors (PAE) shows high confidence as green and low confidence as white.

Figure S8: Secondary antibodies do not display unspecific binding (related to Figure 3 and Figure 5).

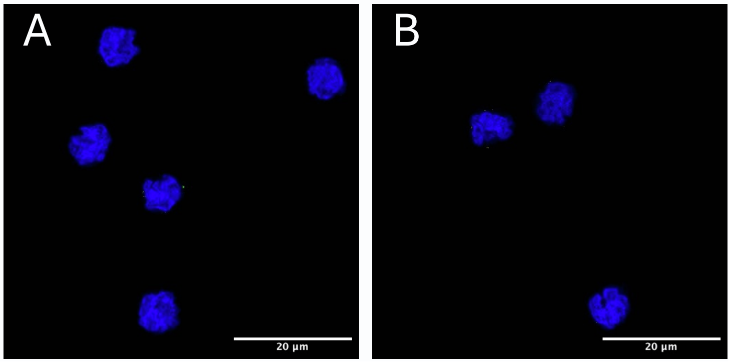

Figure S8: (A) Staining with only anti-human Siglec-5 rabbit polyclonal antibody + secondary antibody goat anti-mouse displayed no unspecific binding. (B) anti-human TLR4 mouse monoclonal antibody + secondary antibody goat anti-rabbit displayed no unspecific binding.

Figure S9: TLR4 inhibition after 4hrs (related to Figure 4).

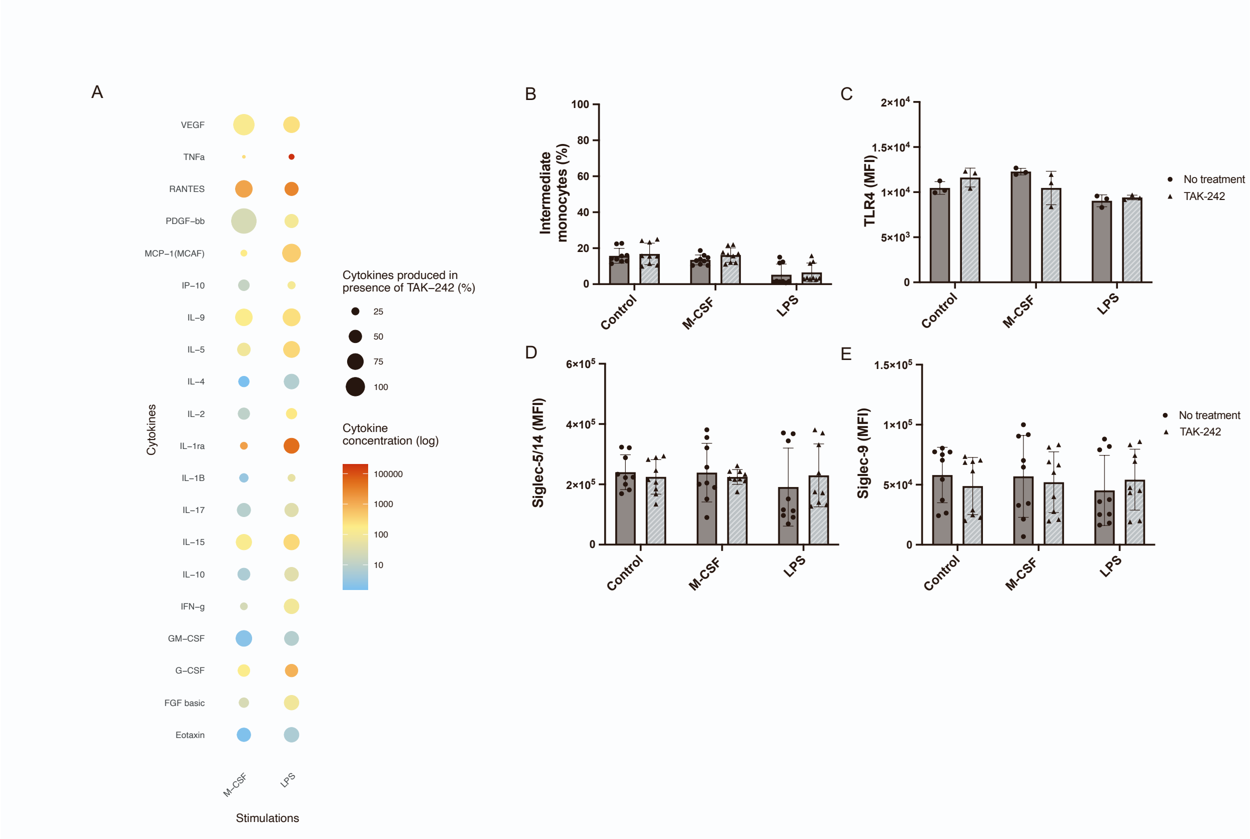

Figure S9: Monocytes were subjected to the TLR4 inhibitor, TAK-242, in combination with M-CSF, LPS, and sialidase for 4hrs. (A) The supernatants of the stimulated monocytes were collected, and a panel of cytokines were screened using the bio-plex assay. Data is analyzed from three biological replicates with three pooled technical replicates (B-E) The effect by TAK-242 on surface receptors was compared to the levels induced by the stimulation without TAK-242. Data is represented as the mean percentage or MFI ± SD of three biological replicates with three technical replicates each. Statistical analysis was performed using two-way ANOVA with Sidak's multiple comparisons test (**p*<0.05; ***p*<0.01; ****p*<0.001; *****p*<0.0001).

Figure S10: TLR4 inhibition does not limit cytokine production in monocytes stimulated with IL1β/TNFα (related to Figure 5).

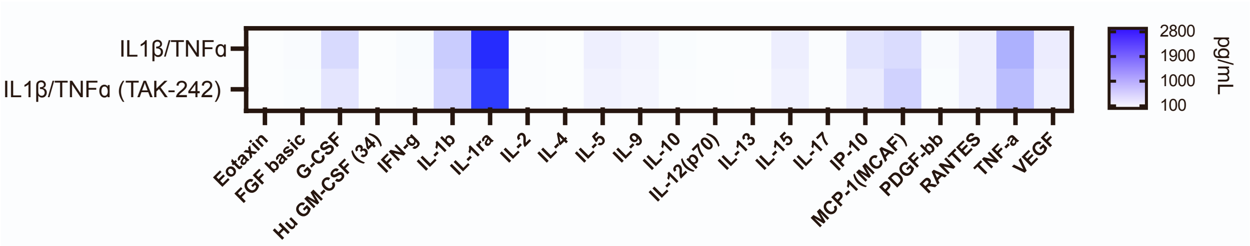

Figure S10: A panel of cytokines were screened using the Bio-Plex Pro Human Cytokine Grp I Panel 27-Plex, after 24hrs of stimulating blood monocytes with IL1β and TNFα in presence and absence of TAK-242, this revealed no strong inhibitory effect by TLR4 inhibition. Three technical replicates where pooled from each biological replicate, three biological replicates were used.

Figure S11: TLR4 inhibition does not affect membrane-expression of Siglec-14 (related to Figure 5).

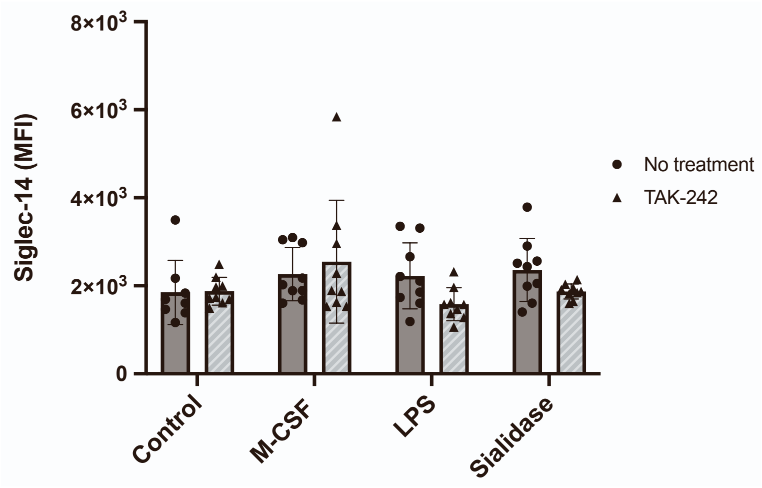

Figure S11: Following 24hrs of incubation in presence or absence of the TLR4 inhibitor TAK-242, no significant difference in membrane-expression of Siglec-14 was observed. Statistical analysis was performed using two-way ANOVA with Sidak’s multiple comparisons test.
